# Position statement on infection screening, prophylaxis, and vaccination of pediatric patients with rheumatic diseases and immunosuppressive therapies, part 3: precautions in situations of surgery, fever, and opportunistic infections

**DOI:** 10.1007/s00431-023-05295-4

**Published:** 2023-12-04

**Authors:** Daniel Clemente, Esmeralda Núñez Cuadros, Marisol Camacho Lovillo, Joan Calzada Hernández, Sara Guillén Martín, Laura Fernández Silveira, María José Lirola Cruz, Alfredo Tagarro, Rosa María Alcobendas Rueda, Agustín López López, Miren Satrustegi Aritziturri, Cristina Calvo

**Affiliations:** 1 Pediatric Rheumatology Unit, Hospital Infantil Universitario Niño Jesús, Madrid, Spain; 2 Pediatric Rheumatology Unit, UGC Pediatría, Hospital Regional Universitario de Málaga, Instituto de investigación biomédica de Málaga (IBIMA), Málaga, Spain; 3 Servicio de Inmunología, Hospital Universitario Virgen del Rocío, Reumatología e Infectología pediátricas, Seville, Spain; 4 Unitat de Reumatologia Pediàtrica, Servei de Pediatria, Hospital Sant Joan de Déu, Esplugues de Llobregat, Barcelona, Spain; 5 Department of Pediatrics, Hospital Universitario de Getafe, CIBERINFEC ISCIII, Carretera de Toledo Km 12, 500, 28905 Getafe, Madrid, Spain; 6 Servicio de Inmunología, Hospital Universitario Virgen del Rocío/Instituto de Biomedicina de Sevilla (IBIS), Reumatología e Infectología pediátricas, Seville, Spain; 7 Department of Pediatric Rheumatology, Instituto Hispalense de Pediatría, Seville, Spain; 8 Pediatrics Department. Hospital Universitario Infanta Sofía, Instituto de Investigación 12 de Octubre (imas12), Universidad Europea, Madrid, Spain; 9 Department of Pediatric Rheumatology, La Paz Children’s Hospital, Madrid, Spain; 10 Department of Paediatrics, Hospital Universitario Puerta de Hierro, Majadahonda, Madrid, Spain; 11 Servicio de Pediatría, Hospital Universitario Donostia, San Sebastián, Spain; 12 Department of Pediatrics, Infectious and Tropical Diseases, Hospital Universitario La Paz, La Paz Research Institute (IdiPaz), Translational Research Network of Pediatric Infectious Diseases (RITIP), CIBERINFEC ISCIII, Madrid, Spain

**Keywords:** Fever, Tuberculosis, Varicella, Herpes zoster, Surgery, Immune-mediated rheumatic diseases, Invasive fungal disease, Consensus

## Abstract

The objective of this study is to provide practical recommendations on the management of pediatric patients with immune-mediated rheumatic diseases receiving immunosuppressive therapies. The recommendations specifically address the cases of surgery, fever, and opportunistic infections (varicella, herpes-zoster, tuberculosis, invasive fungal disease). A qualitative approach was applied. A narrative literature review was performed via Medline. Primary searches were conducted using MeSH terms and free text to identify publications on infections and vaccinations in pediatric patients with immune-mediated rheumatic diseases receiving immunosuppressive therapies. The results were presented and discussed in a nominal group meeting, comprising a committee of 12 pediatric rheumatologists from the Infection Prevention and Treatment Working Group of the Spanish Society of Pediatric Rheumatology. Several recommendations were generated. A consensus procedure was implemented via a Delphi process; this was extended to members of the Spanish Society of Pediatric Rheumatology and Spanish Society of Pediatric Infectious Disease of the Spanish Association of Pediatrics. Participants produced a score ranging from 0 (totally disagree) to 10 (totally agree). Agreement was defined as a vote ≥ 7 by at least 70% of participants. The literature review included more than 400 articles. Overall, 63 recommendations (19 on surgery, fever, and opportunistic infections) were generated and voted by 59 pediatric rheumatologists and other pediatric specialists. Agreement was reached for all 63 recommendations. The recommendations on special situations cover management in cases of surgery, fever, and opportunistic infections (varicella, herpes-zoster, tuberculosis, and invasive fungal disease).

*  Conclusions*: Hereby, we provided consensus and updated of recommendations about the management of special situations such as surgery, fever, and opportunistic in children with immune-mediated rheumatic diseases receiving immunosuppressive therapies. Several of the recommendations depend largely on clinical judgement and specific balance between risk and benefit for each individual and situation. To assess this risk, the clinician should have knowledge of the drugs, the patient’s previous situation as well as the current infectious disease, in addition to experience.

**Table:** 

**What is Known:** *• Infectious diseases and related complications are a major cause of morbidity and mortality in patients with immune-mediated rheumatic diseases.* *• Information on how to manage the treatment in situations of fever, opportunistic infections, and surgery in children is limited, and guidelines for action are often extrapolated from adults.*	
**What is New:** *• In the absence of strong evidence, a literature review and a Delphi survey were conducted to establish a series of expert recommendations that could support the clinical practice, providing a practical and simple day-to-day approach to be used by pediatric rheumatologists.*

**What is Known:**

*• Infectious diseases and related complications are a major cause of morbidity and mortality in patients with immune-mediated rheumatic diseases.*

*• Information on how to manage the treatment in situations of fever, opportunistic infections, and surgery in children is limited, and guidelines for action are often extrapolated from adults.*

**What is New:**

*• In the absence of strong evidence, a literature review and a Delphi survey were conducted to establish a series of expert recommendations that could support the clinical practice, providing a practical and simple day-to-day approach to be used by pediatric rheumatologists.*

## Introduction

Infection and related complications, although uncommon, could be a major cause of morbidity and mortality in patients with immune-mediated rheumatic diseases. The increased risk of infection in this population is probably due to the immune dysregulation of the disease, the use of immunosuppressive drugs, comorbidities, medical/surgical procedures, and frequent clinic visits as described in adults [1].

Pediatric patients with immune-mediated rheumatic diseases could be at higher risk of infection than healthy children because of their underlying condition [2]. The susceptibility to infections, including opportunistic ones, increases further with current intensive treatment strategies incorporating the early use of immunosuppressive therapies such as biologics [3].

Besides, it is important to bear in mind that children are vaccinated during the first years of life and that the immunogenicity of vaccinations may be waned owing to the patient’s immunosuppressed status, thereby further increasing the risk of infection [2]. In addition, the efficiency of vaccines may be reduced when the immunosuppression was already before. It is important to know how to act in the event of an infection in this at-risk population, especially when it comes to infections caused by unusual microorganisms, such as *Mycobacterium tuberculosis* or fungi.

We designed this project to generate practical recommendations on screening for infection, prophylaxis, and vaccination in pediatric patients with immune-mediated rheumatic diseases prior to the initiation of immunosuppressive therapy. Some of the recommendations have been previously published [4]. This article describes contemporary evidence and derived relevant recommendations to guide management of baseline treatment and infections in this group of patients and in special situations such as surgery. This guide intends to esolve questions that may arise the day-to-day clinical practice, thereby improving pediatric patient care and outcomes.

## Methods

This qualitative work was based on a comprehensive narrative literature review, the experience of an expert committee, and the consensus achieved among pediatric rheumatologists. The project adhered to the ethical principles for medical research involving human subjects brought together in the Declaration of Helsinki and was run in accordance with the tenets of Good Clinical Practice. The whole process was supervised by an expert methodologist.

The first stage comprised the selection of a group of 12 pediatric rheumatologists from the Infection Prevention and Treatment Working Group of the Spanish Society of Pediatric Rheumatology (SERPE). Six are also members of the Spanish Society of Pediatric Infections (SEIP). All participants have certified experience in the care of pediatric patients with immune-mediated rheumatic diseases.

## Literature review

With the support of an expert documentalist, a narrative literature review in Medline was performed using PubMed’s Clinical Queries tool, along with individual searches using MeSH and free text terms up to December 2020. The review was then updated for publishing purposes in April 2021. We sought to identify articles describing screening, prophylaxis, precautions in the case of infection or suspicion of infection, and vaccinations in pediatric patients with rheumatic diseases treated with corticosteroids and conventional synthetic and biologic disease-modifying antirheumatic drugs (csDMARDs and bDMARDs). More specific inclusion criteria were as follows: (1) pediatric age (≤ 18 years); (2) publications analyzing any aspects related to screening, prophylaxis, and vaccinations in patients scheduled to begin therapy with corticosteroids, csDMARDs, and bDMARDs; (3) no restrictions concerning the presence or absence of the comparator; (4) type of study (meta-analyses, systematic literature reviews, randomized clinical trials, and observational studies). Two reviewers independently selected the publications, first by title and abstract, then by reading the full paper in detail; finally, they both collected the derived data. Evidence and result tables were generated. Study quality was assessed using the 2011 Oxford scale [5].

## Nominal group meeting

The expert committee held a nominal group meeting, during which they first defined the objectives and users of the document. Through a guided discussion, the experts then argued the available evidence based on the review. They addressed all aspects related to screening before initiation of csDMARDs and bDMARDs, prophylaxis, and management of patients with immune-mediated rheumatic diseases receiving immunosuppressive treatment in the specific cases of surgery, fever, and opportunistic infections (varicella-zoster infections, tuberculosis, and invasive fungal infection). The meeting resulted in the generation of several recommendations.

## Delphi process

With all the previously described information, a series of preliminary recommendations were proposed. After several revisions by the experts, the definitive recommendations were generated and subsequently submitted to an online Delphi vote. In addition, the Delphi process was extended to a group comprising of 92 members of SERPE and SEIP, all experts in their field. Participants voted each recommendation using a scale ranging from 1 to 10 (1 = strongly disagree to 10 = strongly agree). Agreement was defined as a vote ≥ 7 by at least 70% of participants. Recommendations with a level of agreement (LA) below 80% were re-evaluated and, if appropriate, reworded; they then underwent a second round of voting.

## Final consensus document

After the Delphi process and along with the results of the literature review, the final document was drafted. The present document addresses management of patients in cases of surgery, fever, and opportunistic infections (tuberculosis, herpes zoster, virus, and fungi). With the assistance of a methodologist, each recommendation was assigned a level of evidence (LE) and grade of recommendation (GR) according to the recommendations of the Oxford Center for Evidence Based Medicine [5]. The final document was reviewed by the Expert Committee of the Working Group on Prevention of Infections in Children with Rheumatic Diseases of the SERPE, who drafted the final comments.  

## Results

The recommendations generated in this consensus document, as well as the Delphi process results, are depicted in Table 1. A total of 59 experts participated in the Delphi process (response rate 64%): 45 from SERPE and 14 form SEIP.

**Table 1:** Delphi results*

#	Recommendation	Mean	SD	Median	p25	p75	Min	Max	LE	GR	70% ≥ 7
1	In scheduled surgical procedures, methotrexate, and other csDMARDs such as hydroxychloroquine, leflunomide, and sulfasalazine should be continued at the same dosing and schedule	9.21	1.25	10	9	10	5	10	IIIb	D	94%
2	In patients treated with corticosteroids for prolonged periods and at risk of adrenal suppression, the daily dose should be doubled or tripled (24–48 h) before surgery if moderate stress is expected (minor surgery). In situations of severe stress (major surgery), an increase of 3 to 10 times the usual dose would be needed	9.15	1.09	10	8	10	5	10	IIIb	D	98%
3	Maintain treatment with biologics in candidates for surgery, stressing the need for adequate antibiotic perisurgical prophylaxis. Temporarily suspending the drug will be assessed individually in candidates for surgery who are at a high risk of perioperative infectious complications	8.57	2.12	9	8	10	3	10	IIIb	D	87%
4	Febrile syndrome should be managed as in the general population, bearing in mind that follow-up should be closer and that some drugs may partially mask the signs of a potentially serious infection	8.98	3.54	10	9	10	3	10	IIIb	D	91%
5	Patients under treatment with immunosuppressants, including biologics, should undergo a medical evaluation in the case of fever or infection. The recommended approach is as follows: • In the case of mild infections, it is not necessary to withdraw the treatment • In the case of moderate and severe infections, consider suspending treatment until symptoms improve • In cases of persistent activity of the underlying disease or high risk of complications related to the underlying disease, assess maintaining treatment on an individual basis	9.02	1.79	10	9	10	2	10	IIIb	D	94%
6	If treatment is withdrawn, it should be reintroduced when the patient recovers clinical stability and remains afebrile for 24–48 h	9.27	1.27	10	9	10	5	10	V	D	94%
7	Patients undergoing prolonged treatment with corticosteroids and mild stress (e.g., upper respiratory tract infection) should not receive corticosteroid supplementation. In patients with moderate stress (fever > 38 °C, dental extractions, severe vomiting, diarrhea), the attending physician may consider doubling or triple the daily dose	8.26	0.71	8	7	10	3	10	IIIb	D	87%
8	Patients with rheumatic diseases receiving immunosuppressive treatment who have been diagnosed with varicella should generally start intravenous acyclovir in high-risk cases and oral valacyclovir in all other cases	9.23	1.21	10	9	10	4	10	IIIb	D	96%
9	In patients with rheumatic disease receiving immunosuppressive treatment who have been diagnosed with herpes zoster, oral acyclovir or oral valacyclovir is recommended, after individually assessment of whether treatment with intravenous acyclovir is required	9.25	0.96	10	9	10	6	10	IIIb	D	98%
10	In patients with rheumatic disease receiving immunosuppressive treatment who have been diagnosed with varicella or herpes zoster, the immunosuppressive treatment should be interrupted based on the risk of each of the drugs and disease activity and restarted when the infection has completely resolved (all lesions in the crusting stage)	8.94	1.69	10	8	10	2	10	IIIb	D	91%
11	When tuberculosis disease is suspected, the recommended diagnostic procedures are the tuberculin skin test (TST), the interferon gamma release assay (IGRA), imaging tests, and microbiological diagnosis	9.46	1.17	10	9	10	3	10	IIIb	D	96%
12	The treatment for tuberculosis is the same in children with and without rheumatic disease and comprises a 2-month induction phase with 4 drugs (HRZE) and 4 months of maintenance with 2 drugs (HR). In cases of poor adherence, directly observed therapy should be considered	8.85	6.36	10	9	10	1	10	IIIb	D	87%
13	Treatment with biologics should be withdrawn until symptoms are controlled. Treatment with NSAIDs and corticosteroids can be maintained. In cases of significant rheumatic disease activity, csDMARDs can also be maintained	8.94	1.42	9,5	8	10	4	10	IIIb	D	92%
14	If it is necessary to restart biologics, it is recommended to wait until after 6 months of tuberculostatic treatment. If patients require biologics earlier owing to poor control of rheumatic disease, these may be considered after at least 2 months of treatment for tuberculosis and choosing agents with an optimal safety profile in relation to tuberculosis (anakinra, tocilizumab, rituximab, or abatacept). If an anti-TNFα drug must be reintroduced, etanercept is recommended	9.13	1.12	9	9	10	5	10	IIIb	D	96%
15	An infectious diseases specialist with expert knowledge on tuberculosis should be consulted in the case of patients with rheumatic disease and tuberculosis receiving immunosuppressive therapy to rule out extrapulmonary or disseminated disease	9.59	0.89	10	10	10	6	10	IIIb	D	98%
16	Invasive fungal disease should be included in the differential diagnosis of fever especially in patients with prolonged neutropenia caused by immunosuppressive therapy or disease activity	9.46	0.97	10	9	10	5	10	IIIb	D	98%
17	Treatment of an invasive fungal disease will depend on the etiology, although liposomal amphotericin B could be used empirically until the results of additional tests are available	9.22	1.04	10	9	10	6	10	IIIb	D	98%
18	The treatment of choice for *Pneumocystis jirovecii* pneumonia is intravenous trimethoprim-sulfamethoxazole 15–20 mg/kg/day every 6–8 h for 21 days. In addition, systemic corticosteroids should be added in moderate-severe forms with hypoxemia or previous treatment with corticosteroids for another reason	9.41	0.84	10	9	10	7	10	IIIb	D	100%
19	Secukinumab increases the risk of *Candida* infections, although most are mild or moderate and respond to conventional treatment. Cultures should be taken when candidiasis is suspected to rule out the possibility of infection by azole-resistant non-albicans *Candida*	8.89	1.53	9	8	10	4	10	IIIb	D	91%

*SD* standard deviation, *Min* minimum, *Max* maximum, *LE* level of evidence, *GR* grade of recommendation, *DMARDs* disease-modifying antirheumatic drugs, *IGRA* interferon-γ release assay, *TST* tuberculin skin test, *NSAIDs* nonsteroidal anti-inflammatory drugs

^*^The level of evidence was assessed using the Oxford Center for Evidence Based Medicine classification

## Surgery

**Recommendation** **1.** In scheduled surgical procedures, methotrexate, and other csDMARDs such as hydroxychloroquine, leflunomide, and sulfasalazine should be continued at the same dosing and schedule (LE IIIb; GR D; LA 94%).

Methotrexate is the best studied DMARD in the perioperative period, although experience is limited to adults, generally with concomitant conditions. Continuing with methotrexate or other synthetic DMARDs seems safe in the perioperative period, with no adverse effects on healing or the post-surgery infection rate, and reduces the possibility of relapse of joint disease after surgery, too [6, 7].

In patients with systemic lupus erythematosus (SLE), the decision to maintain or discontinue treatments other than hydroxychloroquine (azathioprine, mycophenolate, cyclosporine, and tacrolimus) depends on the severity of SLE, with treatment maintained in cases of severe organ involvement (e.g., lupus nephritis or central nervous system involvement). In mild cases without disease activity, these medications can be suspended 1 week before surgery and restarted 3–5 days after [6].

**Recommendation** **2.** In patients treated with corticosteroids for prolonged periods and at risk of adrenal suppression the daily dose should be doubled or tripled (24–48 h) before surgery if moderate stress is expected (minor surgery). In situations of severe stress (major surgery), an increase of 3 to 10 times the usual dose would be needed (LE IIIb; GR D; LA 98%).

Both clinical evidence and biochemical evidence of adrenal suppression (AS) following discontinuation of therapeutic doses of systemic corticosteroids have been reported in children. Higher doses of corticosteroids, longer-term use, and the timing of administration (evening versus morning) are theoretical risks for this adverse event [8]. Children who have been receiving corticosteroids at pharmacological (supra-physiological) doses for > 2 weeks or multiple short courses of corticosteroid therapy are at risk of adrenal suppression. Prolonged treatments can generate AS up to 12 months after discontinuation [9]. In adults, doses of prednisone > 20 mg/day are considered suppressive, and doses of between 5 and 20 mg are considered to carry risk. Adult patients receiving < 5 mg/day of prednisone are considered not to be at risk for AS [10].

Risk for AS can be assessed prior to surgery or periodically in patients with steroids. Cortisol should be assessed between 7 and 9 am in at-risk patients. A first morning cortisol value of 350 to 500 nmol/L can predict normal hypothalamic–pituitary–adrenal axis function. A first morning cortisol value between 100 and 275 nmol/L suggests possible AS. In this case, consider synthetic ACTH stimulation testing to assist in the diagnosis of AS or administer corticosteroids as replacement therapy during stressful situations.

Stress dosing should be provided for minor or moderate surgery or procedures requiring general anesthesia, with hydrocortisone 50 mg/m^2^ IV (maximum 100 mg) before surgery, followed by 30 mg/m^2^/day to 50 mg/m^2^/day divided every 6 h IV for a further 24–48 h. Cases of most severe stress, such as major surgery, are treated with hydrocortisone 50 mg/m^2^ to 100 mg/m^2^ IV (maximum 100 mg) before surgery, followed by 100 mg/m^2^/24 h IV (maximum 200 mg) divided every 6 h or by continuous infusion. In most instances, stress doses are administered for only 24 to 48 h [8].

**Recommendation** **3.** Maintain treatment with biologics in candidates for surgery, stressing the need for adequate antibiotic prophylaxis. Temporarily suspending the drug will be assessed individually in candidates for surgery who are at a high risk of perioperative infectious complications (LE IIIb; GR D; LA 87%).

In adults, treatment with biologics should be discontinued before elective orthopedic surgeries, based on the intervals of administration of each drug [6]. Some studies suggest that there are fewer complications and local infections in patients who discontinue anti-TNF alpha agents, although this increases the number of flares [11–13].

In children, there are no specific recommendations. Previous experience in clinical practice and retrospective series have not demonstrated an increase in infections in children undergoing surgical procedures. Maintenance of immunosuppressive therapy during surgery in patients with idiopathic uveitis and juvenile idiopathic arthritis (JIA)–associated uveitis did not result in a significant number of infections and was associated with a lower rate of post-surgery uveitis flare [14]. Therefore, suspension is generally not recommended in patients with rheumatic diseases, even not in those who are stable for a long period and in whom the withdrawal of treatment does not entail the risk of a significant flare-up.

Suspension might be considered in patients with a high risk of perioperative infectious complications. To restart biologics, the wound must be healed, the stitches must have been removed, there must be no swelling, erythema or exudates, and no suspicion of local infection, too.

## Fever

**Recommendation** **4.** Febrile syndrome should be managed as in the general population, bearing in mind that follow-up should be closer and that some drugs may partially mask the signs of potentially serious infection (LE IIIb; GR D; LA 91%).

Although it is difficult to generalize in a group of patients as heterogeneous as those with immune-mediated rheumatic diseases, infections are caused mainly by the same microorganisms as in the general population, and therefore this group of patients should be similarly treated [15]. Opportunistic infections, including tuberculosis, are very rare [16].

JIA patients may have an increased risk of bacterial infections, thus a higher possibility of hospital admission. While this appears to be a consequence of the disease itself [17], it may be further worsened by antirheumatic treatment [16], especially with biological therapy [18]. A recent meta-analysis confirmed that anti-TNF therapy slightly—but not significantly—increases the incidence of infection overall compared to non-biological therapies [19].

In patients under treatment with rituximab or tocilizumab, the clinical manifestations of serious bacterial infections may be less recognizable and present without fever and/or low or normal C-reactive protein levels. Therefore, it is necessary to maintain a high index of clinical suspicion, with slightly more meticulous careful management than in the general population, while trying to avoid excess additional tests.

**Recommendation** **5.** Patients under treatment with immunosuppressants, including biologics, should undergo medical evaluation in the case of fever or infection. The recommended approach is as follows:

- In the case of mild infections, it is not necessary to withdraw treatment.
- In the case of moderate and severe infections, consider suspending treatment until symptoms improve.
- In cases of persistent activity of the underlying disease or high risk of complications related to the underlying disease, consider maintaining treatment on an individual basis (LE IIIb; GR D; LA 94%).

Mild infections are defined as those that occur without fever or with low-grade fever, such as upper respiratory tract infections, and resolve with only symptomatic treatment. Moderate infections are considered in the presence of fever (> 38 °C) and/or when medical care is necessary. Infections are considered severe when they require admission to the hospital.

The usual practice is to temporarily suspend or delay the administration of immunosuppressive treatment in cases of severe intercurrent infections, although in the case of mild infections, immunosuppressants may be continued. Moderate infections should be assessed individually depending on the treatment, the timing, and the infection. In certain rheumatic diseases, such as autoinflammatory diseases and vasculitis, a flare of the rheumatic disease may be as serious as or more serious than the infection itself. Therefore, withdrawal should be on an individual basis. Besides, disease activity can manifest as febrile syndrome, and it can be difficult to differentiate a disease flare from an infection.

**Recommendation** **6.** If treatment is withdrawn, it should be reintroduced when the patient recovers clinical stability and remains afebrile for 24–48 h (LE V; GR D; LA 94%).

Immunosuppressive treatment should be restarted once manifestations of the infection have been controlled, appropriate antimicrobial therapy has been established (if necessary), and the patient remains afebrile for 24–48 h. This recommendation is also based on the clinical criteria of experts of maintain the clinical stability of the rheumatic disease as soon as possible, provided that the benefit outweighs the risk and avoiding as far as possible the occurrence of a flare.

**Recommendation** **7.** Patients undergoing prolonged treatment with corticosteroids and mild stress (e.g., upper respiratory tract infection) should not receive corticosteroid supplementation. In patients with moderate stress (fever > 38 °C, dental extractions, severe vomiting, diarrhea), the attending physician may consider doubling or triple the daily dose (LE IIIb; GR D; LA 87%).

Supplemental doses of corticosteroids in patients with mild infections are not recommended. However, stress dosing is recommended in patients at risk of AS with moderate illness (i.e., fever ≥ 38 °C, severe vomiting and/or diarrhea) at 30 to 50 mg/m^2^/day of hydrocortisone equivalent until symptoms resolve. In children on active corticosteroid therapy with doses of ≥ 7.5 mg/m^2^/day of prednisone, stress dosing for moderate illness can be achieved by doubling the therapeutic prednisone dose to be given twice daily (i.e., therapeutic dose is sufficient for stress coverage). When therapeutic corticosteroids are no longer needed, stress dosing should be provided using hydrocortisone. In patients who are unable to tolerate corticosteroids, hydrocortisone must be given parenterally.

In cases of severe illness or injury, the same regimen of hydrocortisone can be administered as in major surgery [8].

## Varicella and herpes zoster

**Recommendation** **8.** Patients with rheumatic disease receiving immunosuppressive treatment who have been diagnosed with varicella should generally start intravenous acyclovir in high-risk cases and oral valacyclovir in all other cases (LE IIIb; GR D; LA 96%).

Treatment with intravenous acyclovir is recommended in immunosuppressed patients diagnosed with varicella, especially those at high risk of developing severe disease (Table 2) Starting treatment within the first 24–48 h of rash onset improves outcome. Oral acyclovir should not be used in the treatment of immunosuppressed children with varicella owing to its low bioavailability. Some experts have used oral valacyclovir, which has better bioavailability than oral acyclovir in selected immunocompromised patients perceived to be at low or medium risk for developing severe varicella (Table 2) [20–22].

**Table 2:** Risk of immunosuppression of varicella with different drugs

Low risk	Medium risk^b^	High risk^c^
Prednisone^a^	Prednisolone/Methylprednisolone: ≥ 40 mg/day for at least a week > 2 mg/kg/day for at least a week Prednisone: ≥ 2 mg/kg/day (20 mg in children ≥ 10 kg) for at least 2 weeks ≥ 1 mg/kg/day at least a month	Biological drugs
Methotrexate^a^	Methotrexate: 10–15 mg/m^2^/week or > 0, 4 mg/kg/week	JAK inhibitors
Azathioprine^a^	Azathioprine ≥ 3 mg/kg/day	
Sulfasalazine	Mercaptopurine ≥ 1.5 mg/kg/day	
Hydroxychloroquine	Cyclosporine, leflunomide, cyclophosphamide	

^a^At lower doses than in the intermediate-risk group

^b^Any of the drugs in the last 3 months

^c^Any of the drugs in the last 6 months

Intravenous acyclovir is administered in children under 2 years of age at 30 mg/kg/day in 3 doses for 7–10 days. In children older than 2 years, especially for those over 12 years, treatment should be at 1500 mg/m^2^/day or 30–45 mg/kg/day in 3 doses for 7–10 days. Valacyclovir should be used in children aged 2 to 17 years at a dose of 20 mg/kg/dose, with a maximum dose of 1000 mg, administered orally 3 times a day for 5 days [23].

In pediatric patients with varicella receiving long-term treatment with corticosteroids, intravenous acyclovir should be administered for 48–72 h, that is, the time when viremia is likely to be present. Thereafter, if the patient is clinically stable, therapy can be completed with orally administered acyclovir [24].

**Recommendation** **9.** In patients with rheumatic diseases receiving immunosuppressive treatment who have been diagnosed with herpes zoster, oral acyclovir or oral valacyclovir is recommended after individual assessment of whether treatment with intravenous acyclovir is required (LE IIIb; GR D; GA 98%).

Oral acyclovir administered within 48–72 h of herpes zoster rash onset reduces the incidence of acute neuritis in healthy adults [25]. Valacyclovir is an alternative antiviral agent with a longer, more convenient dosing interval that may be more effective than acyclovir in treating herpes zoster [26].

In immunosuppressed patients, antivirals should be initiated if vesicles or active lesions are present, regardless of the time since onset. Immunosuppressed patients with uncomplicated herpes zoster infection can also be treated with oral antiviral therapy (valacyclovir or acyclovir); however, those with disseminated, multimetameric, or ophthalmologic herpes zoster infection should be admitted for treatment with intravenous acyclovir. This should be continued for at least 7 days or until all lesions have crusted over and no new lesions have appeared for 48 h [20].

**Recommendation** **10.** In patients with rheumatic diseases receiving immunosuppressive treatment who have been diagnosed with varicella or herpes zoster, the immunosuppressive treatment should be interrupted based on the risk of each of the drugs and disease activity and restarted when the infection has completely resolved (all lesions in the crusting stage) (LE IIIb; GR D; LA 91%).

There are currently no established guidelines to support decision making in this regard. In a retrospective study of children with rheumatic diseases receiving immunosuppressive treatment who had been diagnosed with varicella or herpes zoster, half of the pediatric rheumatologists continued immunosuppressive medication during varicella zoster virus infection. Most of the specialists continued methotrexate in monotherapy but interrupted all therapy when used in combination with biological therapy in two thirds of cases. In addition, the length of the treatment interruption was heterogeneous, 1 to 4 weeks [27].

## Tuberculosis

**Recommendation** **11.** When tuberculosis is suspected, the recommended diagnostic procedures are the tuberculin skin test (TST), the interferon gamma release assay (IGRA), imaging tests, and microbiological diagnosis (LE IIIb; GR D; LA 96%).

The diagnosis of tuberculosis in children is based mainly on clinical and radiological findings, the epidemiological history of contact with adults with tuberculosis (typically bacilliferous), and the positivity of the TST and/or IGRA results. Bacteriological confirmation by isolating *Mycobacterium tuberculosis* in culture is considered the gold standard for diagnosis, although it is difficult to achieve in children (20–50% of cases) and the results may be delayed for several weeks [28, 29].

When tuberculosis is suspected, a simple anteroposterior and lateral chest X-ray should be performed. However, plain radiographs have poor sensitivity for detecting mediastinal and hilar lymphadenopathy, which are the defining feature of primary pulmonary tuberculosis in children. In immunosuppressed patients, the chest X-ray may be normal. In young children with normal plain chest radiography findings but a positive TST and/or IGRA result, a CT scan and/or magnetic resonance imaging can then be useful, which enable earlier and more frequent detection of lymphadenopathy, consolidation, or pleural effusion and for studying extrapulmonary tuberculosis [30, 31].

Traditionally as aforementioned, for the microbiological diagnosis in pulmonary tuberculosis, it is recommended to collect at least three high-quality respiratory samples. In children, it is recommended to collect three fasting gastric juice samples on three consecutive days. The collection of induced sputum (two to four samples) increases diagnostic performance, which is however low in children because they are not usually bacilliferous [32]. In extrapulmonary disease, samples must be obtained from the area of the suspected location, and it is often necessary to perform invasive tests (e.g., biopsy, fine-needle aspiration, lumbar puncture).

Acid-fast staining (Ziehl–Neelsen, auramine) and culture in liquid and solid media should always be performed. Nowadays, genetic amplification techniques (polymerase chain reaction, PCR) are co-adjuvant to smear microscopy and culture isolation. Since they are faster and facilitate diagnosis, they should be used whenever possible. Genetic detection of the most well-known mutations conferring resistance to isoniazid (katG and inhA gene) and rifampicin (rpoB gene) are highly specific and applied. An antibiogram for first-line drugs should be performed on all isolates and extended if resistance is detected [27, 33].

**Recommendation** **12.** The treatment for tuberculosis is the same in children with and without rheumatic diseases and comprises a 2-month induction phase with 4 drugs (HRZE) (isoniazid, rifampicin, pyrazinamide, ethambutol) and 4 months of maintenance with 2 drugs (HR). In cases of poor adherence, directly observed therapy should be considered (LE IIa; GR B; LA 90%).

The treatment of tuberculosis should be similar in patients with or without rheumatic diseases [34]. Considering the rate of resistance to isoniazid (H) in our setting (≥ 4%), the initial guideline of choice without knowing the sensitivity of the strain will be as follows:

- Initial phase (HRZE, 2 months): the fourth most used drug is oral ethambutol (E), with monitoring to detect optic neuritis (visual acuity and distinction between red and green). Duration of the fourth drug: discontinue when the susceptibility of the strain is known (source case). If unknown, maintain for 2 months.
- Maintenance phase (HR, 4 months): in pulmonary tuberculosis due to non-resistant or unknown strains and favorable progress. In cases of extrapulmonary or resistant disease, an infectious diseases specialist should be consulted.

The doses commonly used in the treatment of tuberculosis are detailed in Table 3. In cases of suspected poor adherence, the possibility of directly observed therapy must be considered.

**Table 3:** Dose of first-line tuberculostatic drugs

	Dose in daily regimens, mg/kg/day (dose range)	Maximum daily dose (mg)
Isoniazid (H)	10 (7–15)^a,b^	300
Rifampicin (R)	15 (10–20)^a^	600
Pyrazinamide (Z)	35 (30–40)	2000
Ethambutol (E)	20 (15–25)^c^	2500

^a^Higher doses of isoniazid and rifampicin are used in tuberculous meningitis

^b^Add pyridoxine 15–50 mg/day (maximum 50 mg/day) if exclusively breastfeeding, vegetarians, nutritional disorders, HIV-infected persons, and pregnant adolescents

^c^Ethambutol should be used at more bactericidal doses (20–25 mg/kg/day) during the induction period and decreased to 15–20 mg/kg/day during the maintenance period

**Recommendation** **13.** Treatment with biologics should be withdrawn until symptoms are controlled. Treatment with NSAIDs and corticosteroids can be maintained. In cases of significant rheumatic disease activity, csDMARDs can also be maintained (LE IIIb; GR D; LA 92%).

Biologics should always be withdrawn during the active phase of tuberculosis (2 months). Until the symptoms are controlled, patients with rheumatic diseases can be treated with analgesics and nonsteroidal anti-inflammatory drugs. In severe cases, low-risk DMARDs such as hydroxychloroquine and sulfasalazine can be used. Intra-articular corticosteroids, methotrexate, and cyclosporine are also considered useful and permitted if possible after 2 months of induction.

**Recommendation** **14.** If it is necessary to restart biologics, it is recommended to wait until after 6 months of tuberculostatic treatment. If patients require biologics earlier owing to poor control of rheumatic disease, these may be considered after at least 2 months of treatment for tuberculosis and choosing agents with an optimal safety profile in relation to tuberculosis (anakinra, tocilizumab, rituximab, or abatacept). If an anti-TNFα drug must be reintroduced, etanercept is recommended (LE IIIb; GR D; LA 96%).

The Italian multidisciplinary task force for screening of tuberculosis before and during biological therapy (SAFEBIO) considers that biologics can be restarted after 6 months of treatment, which is usually the time to complete the full course of tuberculostatic treatment [35].

In patients with a high rheumatic disease activity, low-risk biologics for tuberculosis can be used 2 months after completing induction therapy. Anakinra, tocilizumab, rituximab, and abatacept can be considered. When a TNF inhibitor is required, etanercept should be chosen instead of monoclonal antibodies [36]. Both sirolimus and JAK inhibitors carry some risk, although data on the latter remain very limited. While these recommendations are for adults, for the time being, it seems reasonable to be implemented in children.

New, shorter regimens (4 months) have been demonstrated to be useful in children with non-severe tuberculosis, but they have not been tested in immunosuppressed children yet. Shorter treatments might be helpful in children with non-severe tuberculosis and active disease needing biologics [37].

**Recommendation** **15.** An infectious diseases specialist with expert knowledge on tuberculosis should be consulted in the case of patients with rheumatic disease and tuberculosis receiving immunotherapy to rule out extrapulmonary or disseminated disease (LE IIIb; GR D; LA 89%).

A thorough evaluation is required to rule out extrapulmonary or disseminated tuberculosis, especially in patients receiving biologics. Although few pediatric patients develop *Mycobacterium tuberculosis* infection, likely owing to effective screening, in patients with JIA, extrapulmonary involvement seems to be more frequent than pulmonary involvement [38]. In addition, resistance to tuberculosis treatment—even first-line drugs—is increasingly common [39]. It is therefore advisable to consult a specialist in infectious diseases to achieve a thorough investigation and ensure optimal treatment.

## Invasive fungal disease

**Recommendation** **16.** Invasive fungal disease should be included in the differential diagnosis of fever especially in patients with prolonged neutropenia caused by immunosuppressive therapy or disease activity (LE IIIb; GR D; LA 98%).

Invasive fungal disease (IFD) should be considered in patients with pronounced immunosuppression or in critically ill patients, those with hematological-oncological diseases, or undergoing hematopoietic stem cell transplants, patients with prolonged admissions to intensive care units, and those with severe primary or acquired immunodeficiencies [40, 41].

While not a common complication in rheumatic diseases, IFD has been described in adults with rheumatoid arthritis [42] and, especially, SLE. Associated risk factors include the presence of prolonged neutropenia, which may be caused by immunosuppressive treatment or by high disease activity. Therefore, IFD should be included in the differential diagnosis of patients with SLE undergoing immunosuppressive treatment who present with central nervous system involvement, atypical skin manifestations, or pulmonary infiltrates [43, 44].

IFD have rarely been described in children with autoimmune diseases (JIA, uveitis, Crohn’s disease) treated with anti-TNF agents. The risk seems to be greater with infliximab than with other anti-TNFs; besides, the use of concomitant corticosteroids is a predictor of severity. Most cases have been reported in North America, mainly due to *Histoplasma* species [45]. Isolated cases of IFD have also been described with rituximab and anakinra [46].

**Recommendation** **17.** Treatment of an invasive fungal disease will depend on the etiology, although liposomal amphotericin B could be used empirically until the results of additional tests are available (LE IIIb; GR D; LA 98%).

Both caspofungin and liposomal amphotericin B are recommended for empirical treatment in patients with hematological-oncologic diseases and febrile neutropenia, although amphotericin B appears to be the first choice [47]. When an IFD is confirmed and the etiology is known, treatment can be targeted. First-line therapy for candidemia consists of either fluconazole, caspofungin, or liposomal amphotericin B, with voriconazole being first-line therapy for invasive aspergillosis. Histoplasmosis and cryptococcosis are both treated with liposomal amphotericin B [48].

**Recommendation** **18.** The treatment of choice for *Pneumocystis jirovecii* pneumonia is intravenous trimethroprim-sulfamethoxazole 15–20 mg/kg/day every 6–8 h for 21 days. Systemic corticosteroids should be added in moderate-severe forms with hypoxemia or previous treatment with corticosteroids for another reason (LE IIIb; GR D; LA 100%).

*Pneumocystis jirovecii* pneumonia (PCP) can affect immunocompromised patients despite the use of antimicrobial prophylaxis. Mortality associated with PCP continues to be high; therefore, it is important to maintain a high level of suspicion, as early initiation of treatment is an important prognostic factor [49].

The treatment of choice of *Pneumocystis jirovecii* pneumonia is trimethroprim-sulfamethoxazole (TMP-SMX) by intravenous administration until patients are clinically stable (e.g., no respiratory distress or hypoxemia) and have a functioning gastrointestinal tract. The dose of TMP-SMX is 15 to 20 mg/kg (dosing is based upon the TMP component and expressed as mg/kg per day of TMP) in three or four divided doses. Alternatives for TMP-SMX are clindamycin (40 mg/kg/day every 6 h) plus primaquine (0.3 mg/kg/day) or atovaquone (30–40 mg/kg/day). All these agents can be given orally. While early clinical deterioration (within the first 3–5 days after treatment initiation) is common, the response to therapy should not be re-evaluated before 8 days of full-dose treatment. In patient with clinically documented treatment failure at day 8, a repeat bronchoscopy and bronchoalveolar lavage (BAL) to look for co-infections should be ordered [50].

Corticosteroids should be added in patients with moderate or severe pneumonia (dyspnea at rest or at minimal exertion, arterial oxygen saturation < 95%) or with previous treatment with corticosteroids, preferably starting within 72 h of antibiotic initiation. Prednisone or methylprednisolone 1–4 mg/kg/day are generally used, tapering until the 21-day therapy has been completed.

csDMARDs and bDMARDs should be discontinued as soon as PCP is suspected. DMARDs can be restarted after complete resolution of the infection, maintaining secondary prophylaxis with TMP-SMX, especially if lymphopenia persists [51].

**Recommendation** **19.** Secukinumab increases the risk of *Candida* infections, although most are mild or moderate and respond to conventional treatment. Cultures should be taken when candidiasis is suspected to rule out the possibility of infection by azole-resistant non-albicans *Candida* (LE IIIb; GR D; GA 96%).

IL-17-mediated immunity is a fundamental mechanism to protect the mucous membranes and skin from fungal infections [52]. Therefore, *Candida* infections have been described in adult patients with moderate-severe psoriasis and/or psoriatic arthritis treated with IL-17 inhibitors (secukinumab, ixekizumab, and bromalizumab). Most infections are superficial and localized. Topical treatment with azoles is generally effective and safe, and it is not necessary to discontinue immunosuppressive therapy. The risk of systemic dissemination is low. Cultures of blood, urine, and skin should be taken when candidiasis is suspected to rule out the possibility of infections by azole-resistant non-albicans *Candida* [53, 54].

In pediatric patients, secukinumab is indicated only in children older than 6 years with psoriasis. However, several ongoing trials have reported favorable results for psoriatic JIA and enthesitis-related arthritis. This may increase the use of secukinumab in the coming years [55].

## Discussion

Few studies compare the risk of infection between pediatric patients with rheumatic diseases and healthy children, and even fewer make comparisons between specific diseases or individual drugs. However, some have shown an increased risk of infections, especially serious and opportunistic infections, in children receiving immunosuppressive treatment. Therefore, there is a need to establish clear recommendations for management of children with suspected infection who are about to undergo a scheduled surgical procedure.

Based on the best available evidence, we present a series of recommendations concerning the management of patients with immune-mediated rheumatic diseases with immunosuppressive treatment in the case of surgery, fever, and opportunistic infections.

For this purpose, we followed the nominal group and Delphi methodologies, which are widely used to prepare this type of document. In addition, along with a review of the available evidence, a group of experts in the field was selected for the drafting of the recommendations.

Several of the recommendations rely largely on the clinical judgement and specific balance of the risk to benefit ratio for each individual and situation. For this risk assessment, the clinician should have evidence-based knowledge of the drugs, details of the previous patient history, and the current infectious disease, as well as experience. If needed, the attending physician should search for additional consultation provided by experienced colleagues.

The recommendations are intended to assist specialists in their routine care of pediatric patients with rheumatic disease. In addition, there is no doubt that the availability of explicit recommendations for immunosuppressive treatments in the real-world setting is an essential element of good clinical practice, as demonstrated in this document. Data from pharmacovigilance cohorts will be useful for actual risk monitoring in clinical practice [3]. Periodic update of the current recommendations will align and support a contemporary good clinical practice.
